# Effects of the *sliaa9* Mutation on Shoot Elongation Growth of Tomato Cultivars

**DOI:** 10.3389/fpls.2021.627832

**Published:** 2021-05-20

**Authors:** Chihiro Abe-Hara, Kohji Yamada, Naoki Wada, Risa Ueta, Ryosuke Hashimoto, Keishi Osakabe, Yuriko Osakabe

**Affiliations:** ^1^Faculty of Bioscience and Bioindustry, Tokushima University, Tokushima, Japan; ^2^School of Life Science and Technology, Tokyo Institute of Technology, Yokohama, Japan

**Keywords:** parthenocarpy, photomorphogenesis, elongation growth, shoot growth, auxin, SlIAA9, CRISPR-Cas9, tomato

## Abstract

Tomato *INDOLE-3-ACETIC ACID9* (*SlIAA9*) is a transcriptional repressor in auxin signal transduction, and *SlIAA9* knockout tomato plants develop parthenocarpic fruits without fertilization. We generated *sliaa9* mutants with parthenocarpy in several commercial tomato cultivars (Moneymaker, Rio Grande, and Ailsa Craig) using CRISPR-Cas9, and null-segregant lines in the T1 generation were isolated by self-pollination, which was confirmed by PCR and Southern blot analysis. We then estimated shoot growth phenotypes of the mutant plants under different light (low and normal) conditions. The shoot length of *sliaa9* plants in Moneymaker and Rio Grande was smaller than those of wild-type cultivars in low light conditions, whereas there was not clear difference between the mutant of Ailsa Craig and the wild-type under both light conditions. Furthermore, young seedlings in Rio Grande exhibited shade avoidance response in hypocotyl growth, in which the hypocotyl lengths were increased in low light conditions, and *sliaa9* mutant seedlings of Ailsa Craig exhibited enhanced responses in this phenotype. Fruit production and growth rates were similar among the *sliaa9* mutant tomato cultivars. These results suggest that control mechanisms involved in the interaction of AUX/IAA9 and lights condition in elongation growth differ among commercial tomato cultivars.

## Introduction

Tomato (*Solanum lycopersicum*) – a model horticultural crop – is one of the most important vegetable and fruit crops worldwide. In wild-type tomato plants, fruiting can be induced by artificial pollination and the application of exogenous auxin. Parthenocarpy, which allows fruit production without fertilization, is a hugely important agricultural trait and has been produced by traditional breeding in limited species and cultivars. The fruiting of tomato is controlled by auxin signaling factors, tomato Auxin Responsive Factor 8 (SlARF8), and the auxin responsive transcription repressor INDOLE-3-ACETIC ACID 9 (SlIAA9; Wang et al., 2005; Goetz et al., 2006). SlIAA9 has four conserved domains and two functional motifs (Wang et al., 2005; Saito et al., 2011). Loss-of-function mutants of *SlIAA9* in tomato exhibit parthenocarpy – a desirable trait in tomato – where fruit set and growth are stimulated without fertilization; parthenocarpy can overcome problems with fruit setting under unfavorable environmental conditions. The *sliaa9* mutant tomatoes generated by ethyl methanesulfonate (EMS) treatment of several cultivars, such as Micro-Tom, Ailsa Craig, M82, and Red Setter, showed altered leaf morphology (Zhang et al., 2007; Berger et al., 2009; Saito et al., 2011; Mazzucato et al., 2015). In Micro-Tom, the *AS-IAA9* line (*SlIAA9* downregulated-line) exhibited phenotypic changes in leaf morphology and parthenocarpy (Wang et al., 2005). Hypersensitivity to auxin, enhanced hypocotyl/stem elongation, reduced apical dominance, and upregulation of *SlIAA3* expression were also detected in the *AS-IAA9* line (Wang et al., 2005). We have previously reported the generation of *sliaa9* knockout mutants using the clustered regularly interspaced short palindromic repeat (CRISPR)-CRISPR-associated protein-9 nuclease (Cas9) system; the *sliaa9* mutant showed altered leaf morphology and parthenocarpy (Ueta et al., 2017). In the CRISPR-Cas9 system, the Cas9 nuclease and a guide RNA (gRNA) form a complex and induce a double-strand break (DSB) at the target site. The CRISPR-Cas9 system, which is used to generate target gene-specific mutations, has been applied in many plant species to create novel useful crops by molecular breeding (Osakabe and Osakabe, 2015).

Auxin affects various growth and developmental processes in plants. The relationship between the auxin signal transduction pathway and other environmental factors, such as light conditions, has not been well studied in commercial tomato cultivars. Light conditions lead to various effects in auxin responses (Halliday et al., 2009). When young plants grow in shade, hypocotyl elongation known as the shade avoidance response is induced, in which auxin synthesis is enhanced by PHYTOCHROME B (PHYB) under low red: far red (R:FR; shade) or low light conditions (Trupkin et al., 2014; Ballaré and Pierik, 2017). On the contrary, phytochrome A (PHYA) suppresses shade avoidance responses in plants (Ganesan et al., 2012). It has been suggested that several AUX/IAAs interact with, and are phosphorylated by, PHYA, which inhibits AUX/IAA degradation. As a result, the negative regulation of shade avoidance responses (i.e., inhibition of hypocotyl elongation) is induced under shade (Colón-Carmona et al., 2000; Yang et al., 2018; Ma and Li, 2019). Cryptochrome 1 (CRY1) also interacts with AUX/IAA and negatively regulates auxin signaling in *Arabidopsis* (Xu et al., 2018).

It is important to clarify the effects of light environments on crop growth for agricultural applications. In this study, we analyzed shoot growth of *sliaa9* knockout mutants of three commercial tomato cultivars (Ailsa Craig, Moneymaker, and Rio Grande), which were generated using CRISPR-Cas9, under different light intensity. Null-segregants in next generation progenies of the mutants were isolated, and we demonstrated an effect of normal or low light conditions on shoot growth of *sliaa9* mutants. They exhibited different shoot growth phenotypes and photomorphogenesis, although they showed parthenocarpy in common. These results suggest that various control mechanisms involved in tomato shoot growth in commercial cultivars are mediated by AUX/IAA9 and light.

## Materials and Methods

### Plant Materials and Growth Conditions

Three varieties of *Solanum lycopersicum* L., i.e., cv. Moneymaker, Rio Grande, and Ailsa Craig, were used in this study. Moneymaker and Ailsa Craig are indeterminate type and Rio Grande is a determinate type tomato. Tomato seeds were sterilized by 70% EtOH and 10% sodium hypochlorite solution (Antiformin, Wako, Japan) including 0.005% Triton X-100 (octylphenol ethoxylate, Wako, Japan). Sterilized seeds imbibed water overnight and were sown on germination medium [1.5% sucrose, 1/2 × MS (pH 5.7)]. Expanded cotyledons 7 days after sowing were used for transformation. Tomato calli and shoots were grown in tissue culture in a growth chamber under conditions of 21–25°C with 15 h light at 30–50 μmol m^−2^ s^−1^/9 h dark. T1 or T2 tomato plants imbibed water overnight, germinated in Jiffy-7 (Jiffy), and grown in a growth chamber (CLE-305, TOMY, Japan) or plant growing rack (CR-400L, TOMY, Japan) under conditions of 21–25°C with 15 h light at 40–70 μmol m^−2^ s^−1^ (low light conditions) or 150–250 μmol m^−2^ s^−1^ (normal light condition)/9 h dark.

### CRISPR-Cas9 Vector

In our previous study (Ueta et al., 2017), the *SlIAA9* (*Solyc04g076850*) target sequence, 5'-GAGCTCAGGCTCGGTCTACC-3' (gRNA2) was used for genome editing of all four tomato cultivars. The CRISPR-Cas9 expression vector, pEgP237-2A-GFP (Ueta et al., 2017; Supplementary Figure S1A), was used, which comprises a gRNA expression cassette under the control of the *Arabidopsis U6 snRNA-26* (*AtU6-26*) promoter, and a Cas9 expression cassette for an *Arabidopsis* codon-optimized spCas9 (AtCas9) fused to GFP *via* a 2A peptide, which is driven by the 2 × *CaMV35SΩ* promoter. The AtCas9 contains the 3 × NLS at its C-terminus. In this vector, a kanamycin-resistance marker was used for selection of transgenic plants.

### Transformation of Tomato Plants

The CRISPR-Cas9 expression vector was introduced into *Agrobacterium tumefaciens* (GV2260) and used to transform tomato as described previously (Ueta et al., 2017; Hashimoto et al., 2018). Briefly, *Agrobacterium* harboring CRISPR-Cas9 vector was cultured on LB medium or AB-MES medium. Leaf discs prepared from cotyledons were subjected to *Agrobacterium* infection. After co-culture for several days in the dark, the leaf discs were transferred to callus induction medium (CIM) 1 (3% sucrose, 1 × MS, 100 mg/L kanamycin, 1.5 mg/L trans-zeatin, and 25 mg/L meropenem) and cultured for 4 weeks for Moneymaker and Ailsa Craig. After the appearance of calli, shoot induction medium (SIM) 1 (3% sucrose, 1 x MS, 100 mg/L kanamycin, 1.0 mg/L trans-zeatin, and 25 mg/L meropenem) was used for further incubation. Leaf discs from Rio Grande were incubated in CIM2 (3% sucrose, 1 × MS, 50 mg/L kanamycin, 1 mg/L BA, 0.1 mg/L IAA, and 25 mg/L meropenem) for 4–5 weeks after co-culture, then calli were transferred to SIM2 (3% sucrose, 1 × MS, 50 mg/L kanamycin, 1.0 mg/L trans-zeatin, 0.5 mg/L IAA, and 25 mg/L meropenem) and incubate for at least a further 5 weeks. Transgenic shoots were transferred to root induction medium (1.5% sucrose, 0.5 × MS, 50 mg/L kanamycin, and 25 mg/L meropenem) for 2–4 weeks and then to soil pots.

### Mutation Analyses in CRISPR-Cas9 Target Sites

Genomic DNA was isolated from tomato leaves using Nucleospin Plant II (TaKaRa, Japan), and used for amplification of target sequences by PCR using PrimeSTAR GXL DNA Polymerase (TaKaRa, Japan). An *Acc*I site is located on the *SlIAA9* target sequence, and PCR-RFLP was used for detection of the mutation. In PCR-RFLP, PCR fragments were digested with *Acc*I (NEB, Japan) and analyzed by agarose gel electrophoresis. For Sanger sequencing analysis, PCR fragments purified from agarose gels were cloned by the Seamless ligation cloning extract (SLiCE) method (Motohashi, 2015) into cloning vector pNEB193 (NEB, Japan). All primers used for PCR are listed in Supplementary Table S1.

### PCR for Detection and Southern Blot Analysis of Null-Segregant Plants

Around 20–50 plants of the T1 generation and eight plants of the T2 generation were selected randomly for PCR-based detection of T-DNA regions. Preparation of total DNA was as described above. Nine regions in the T-DNA in the CRISPR-Cas9 vector were selected and amplified using PrimeSTAR GXL polymerase (TaKaRa, Japan). All primers used for PCR and the corresponding amplicon sizes are indicated in Supplementary Table S1. PCR experiments were replicated three times for each line.

For Southern blot, total DNA was extracted from leaves of WT and *sliaa9* mutants by the modified cetyltrimethylammonium bromide (CTAB)-based DNA extraction method (Murray and Thompson, 1980). Briefly, tomato leaves were ground in liquid nitrogen and incubated in 2% CTAB solution [100 mM Tris-HCl (pH8.0), 20 mM EDTA (pH8.0), 1.4 M NaCl, and 2% (w/v) CTAB]. The lysates were extracted with chloroform:isoamyl alcohol (24:1), and then 1% CTAB solution [50 mM Tris-HCl (pH8.0), 10 mM EDTA (pH8.0), 1% (w/v) CTAB] was added to the water layer. Total DNAs were obtained by CsCl-EtOH precipitation. Total DNA was digested with *Hpa*I, which has a single restriction recognition site in the vector (Supplementary Figure S1A), fractionated on 1% agarose gels, and transferred to nylon membrane (Zeta-Probe, Bio-Rad, United States) by capillary transfer. After UV cross-linking (UVP CL-1000 crosslinker, Analytik Jena, Upland, CA, United States), the membranes were hybridized with DIG-labeled probes for *gRNA*, *Cas9*, and *LHCB* (Solyc02g070970) genes. Probes were amplified using pEgP237-2A-GFP (for gRNA and Cas9) or tomato genomic DNA (for *LHCB*) using the primers listed in Supplementary Table S1 and labeled with DIG using a DIG High Prime DNA Labeling and Detection Kit (Roche, Basel, Switzerland). Probe labeling, membrane hybridization, and detection were carried out according to the manufacturer’s instructions. Hybridization signals were detected and imaged with PXi (SYNGENE, Bangalore, India).

### Off-Target Analysis by Amplicon Sequencing Using MiSeq

Amplicon sequencing was performed using MiSeq (Illumina, Japan) and MiSeq Reagent Kit v2 Nano (Illumina, Japan) was used for analysis. Genomic DNAs were used to amplify the region of the CRISPR-Cas9 target sites or off-target sequences by PCR. One off-target candidate sequence (SL2.50 ch6:26946923-26946946) was selected by Cas-OT to examine the mutation. PCR products were separated by electrophoresis, purified from the agarose gel using the Wizard SV Gel and PCR Clean-Up System (Promega, Japan), and used as templates for a second round of PCR. Second PCR was performed using TruSeq HT primer (Illumina, Japan). All primers used for PCR are listed in Supplementary Table S1. MiSeq data were analyzed using CLC Genomics Workbench software version 7.5.1 (CLC bio, Japan) and mapped on the off-target candidate using Integrative Genomics Viewer (IGV; Broad Institute).

### Plant Growth and Leaf Measurements

Shoot lengths of wild-type and *sliaa9* mutants under low or normal light conditions were measured until 48 days after germination. Hypocotyl length in young seedlings of the *sliaa9* mutants and wild-type were measured at 21 days after germination. The third to tenth internode length of the *sliaa9* mutants and the wild-type were measured at 48 days after germination. Several parameters of the fifth to tenth matured leaves from each plant of the *sliaa9* mutants and the wild-type were measured 48 days after germination. The parts of leaves measured are shown in Supplementary Figure S4A. Leaf area was measured using ImageJ. Average values were determined using 2–6 lines of the same mutant allele. All experiments repeated 2–3 times.

### Measuring Leaf Chlorophyll Concentration in Tomato

SPAD-502Plus (Konica Minolta, Japan) was used to measure leaf chlorophyll concentration in leaves. SPAD values were measured at 3–8 sites near the principal vein on the wide part of the tomato leaf on the third or higher fully elongated leaves. In total, 20–30 SPAD values in a plant were analyzed; average values were determined using 3–6 lines of the same mutant allele. All experiments repeated three times.

### qRT-PCR

Hypocotyls of 21-days-old seedlings and the third internodes of 35-days-old seedlings grown under low or normal light intensity were harvested, respectively. Total RNAs were extracted using RNeasy Plant Mini Kit on the QIAcube automated system (Qiagen), followed by reverse-transcribed using a ReverTra Ace qPCR RT Master Mix with gDNA remover (TOYOBO) following the manufacturer’s instructions. Quantitative PCR was performed with GoTaq qPCR Mater Mix (Promega) on LightCycler 96 (Roche). The expression levels of *SlIAA3* (5'-GCAATTCAAATCAATCATTCTTTCT-3' and 5'-GTTTATTATCCCAGGCAAACCTAAT-3') were normalized relative to those of a reference gene, *Slactin7-like* (5'-TGTCCCTATCTACGAGGGTTATGC-3' and 5'-AGTTAAATCACGACCAGCAAGAT-3'; Hashimoto et al., 2018). Relative expression was calculated by two to the power of relative expression (log_2_), calculated by subtracting Ct values of *IAA3* from those of *Slactin7-like*.

## Results

### Detection of *SlIAA9* Mutations in Commercial Tomato Cultivars

**Figure 1:**
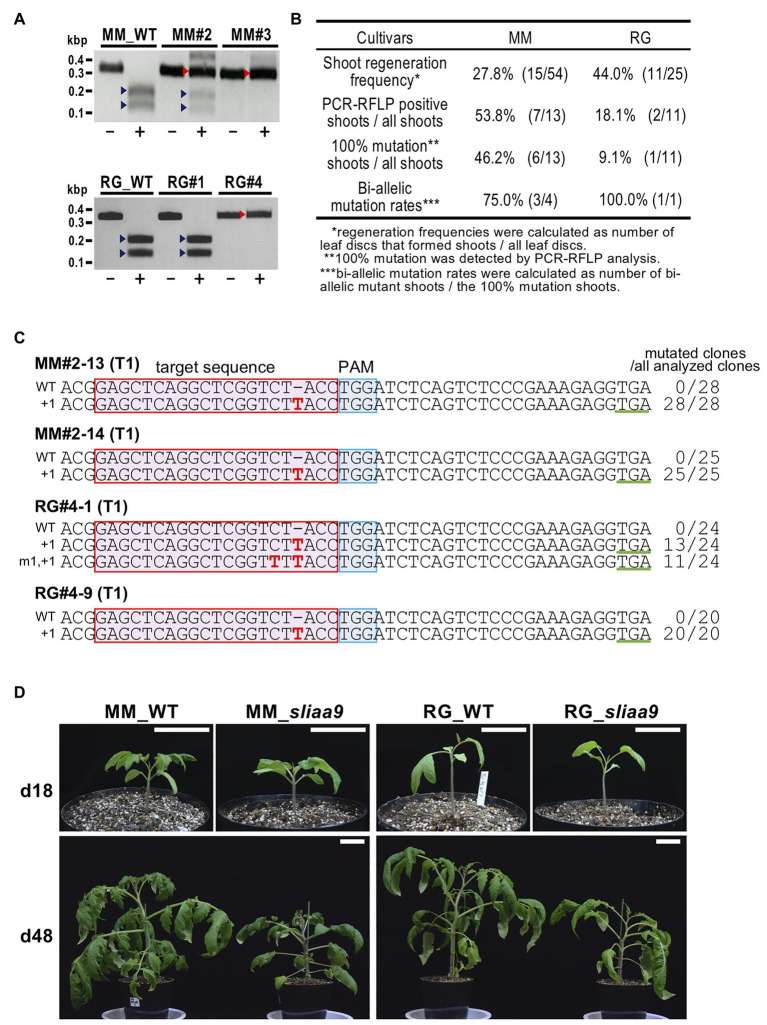
Isolation of *sliaa9* Knockout Mutants in Various Tomato Cultivars. **(A)** PCR-RFLP analysis of *SlIAA9* in CRISPR-Cas9 transgenic T0 lines (MM#2, MM#3, RG#1, and RG#4). Target sites in the *SlIAA9* gene were amplified by PCR using total DNA isolated from T0 shoots of cultivars Moneymaker and Rio Grande. PCR products were digested with *Acc*I, which has a recognition site at the CRISPR-Cas9 target site. MM; Moneymaker, RG; Rio Grande, −; untreated, +; treated with *Acc*I, red arrowheads; mutated bands, blue arrowheads; wild-type fragments digested with *Acc*I. **(B)** Mutation efficiency in regenerated shoots (T0). **(C)** *SlIAA9* mutation sequences in *sliaa9* T1 lines (MM#2-13, MM#2-14, RG#4-1, and RG#4-9) were analyzed by Sanger sequencing. MM; Moneymaker, RG; Rio Grande, red box; target sequence, blue box; PAM sequence, green underline; newly generated stop codon following CRISPR-Cas9 editing, red characters; and mutated nucleotides. The wild-type (WT) sequences are on top in each alignment. The numbers on the left of the sequences indicate mutated nucleotides. The number of clones analyzed is shown to the right of the sequences. **(D)** Wild-type and the representative *sliaa9* T2 lines 18 days and 48 days after germination. The plants were grown under low light conditions. Left; MM (Moneymaker; T2 line, MM#2-13-4), right; RG (Rio Grande; T2 line, RG#4-1-6). Bars = 5cm.

A CRISPR-Cas9 vector targeting exon 2 of the *SlIAA9* gene (+217 to +236 from ATG, 5'-GAGCTCAGGCTCGGTCTACC-3'), which was used to generate mutants in tomato cultivars, Micro-Tom and Ailsa Craig, in a previous study (Ueta et al., 2017), was introduced into Moneymaker and Rio Grande *via Agrobacterium* transformation. PCR-RFLP analysis using an *Acc*I restriction enzyme site located at the CRISPR-Cas9 target was performed to detect CRISPR-Cas9-induced mutations in the T0 regenerated tomato shoots. Non-digested fragments in PCR-RFLP analysis indicated 100% somatic mutations in all cultivars (Figures 1A,B). The progenies of *sliaa9* T0 100% mutant lines (T1 lines) were isolated by self-pollination from T0 mutant lines. Sanger sequencing analysis of cloned PCR fragments at the target sites in the T1 mutant lines revealed only one or two types of mutation, which were found at similar levels in all the sequenced clones in these lines (Figure 1C; Supplementary Figure S1). These results suggest the generation of bi-allelic mutants, in which the *SlIAA9* mutations introduced a frame shift just after domain I, and a stop codon was generated to produce truncated SlIAA9. The progenies of *sliaa9* knockout mutant lines (T1 mutants) were isolated by self-pollination from T0 mutant lines, and T1 mutants showed homozygotes or heterozygotes that were segregated from the parental T0 generation (Supplementary Figure S1). Since T1 mutants were generated from T0 lines by self-pollination, the T-DNA, including the CRISPR-Cas9 cassette, can be segregated out. To detect T-DNA insertion in the genomic DNA of *sliaa9* T1 progenies, PCR analysis designed to amplify nine T-DNA regions and Southern blot analysis were performed using specific primers and DIG-labeled probes, respectively (Supplementary Table S1; Supplementary Figure S1A). Several null-segregant mutants were isolated, in which no T-DNA regions were detected by PCR or Southern blot analysis (Supplementary Figures S1B,C). T-DNA regions were also undetected in the T2 progenies of MM#2, i.e., MM#2-13 (Supplementary Figure S1B). An off-target candidate sequence was analyzed using next generation sequencing, Miseq, and the results suggest that off-targets were not detected in mutant plants (Supplementary Figure S2). The T2 progenies of these null-segregant *sliaa9* T1 mutant lines MM#2-13, RG#4-1, and AC#1-1 were named as MM-*sliaa9*,” “RG-*sliaa9*,” and “AC-*sliaa9*,” respectively, and used for subsequent experiments.

### Altered Elongation Growth in *SlIAA9* Knockout Mutants

**Figure 2:**
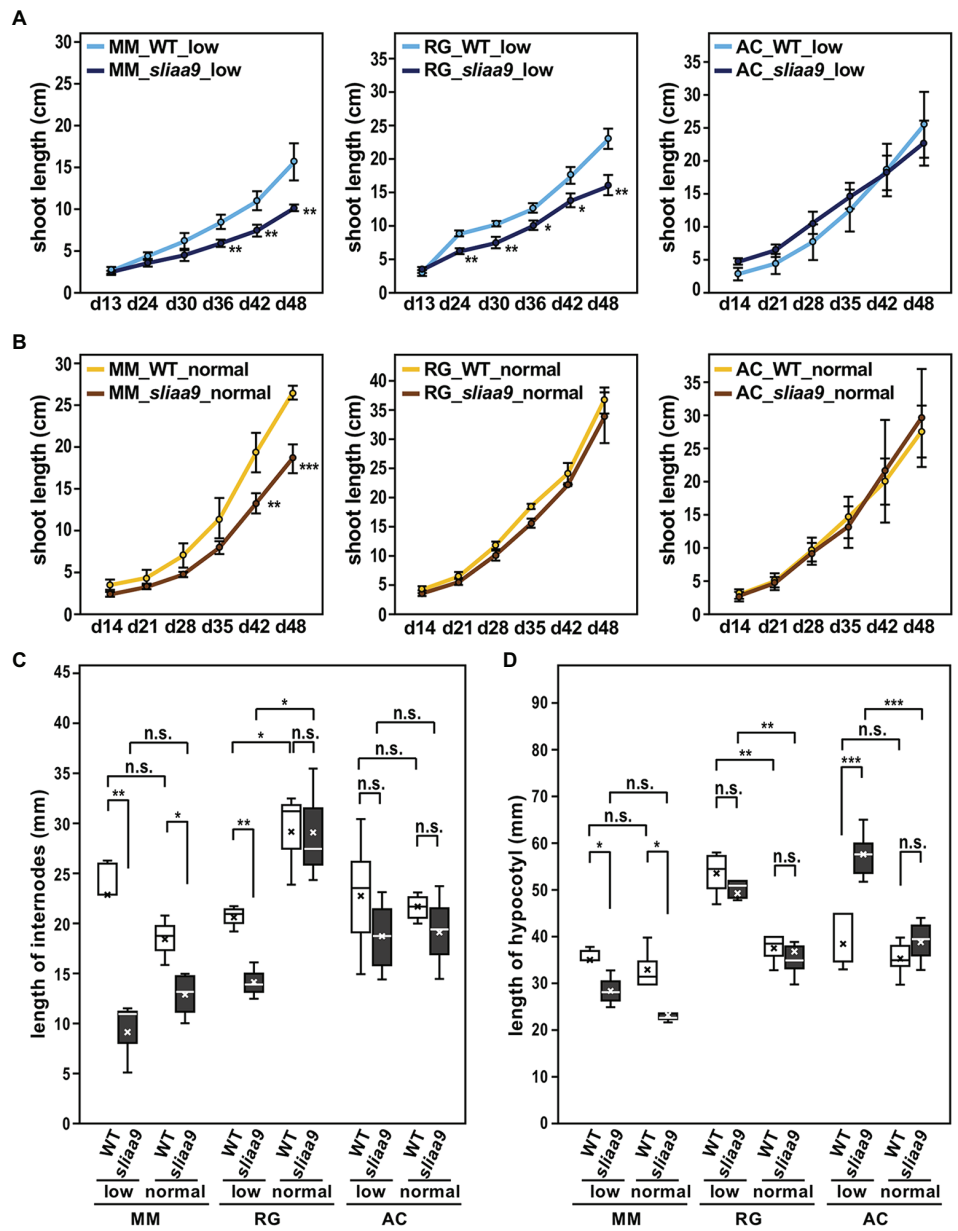
Elongation growth of stem/hypocotyl in the *sliaa9* mutants under different light conditions. **(A)** Shoot growth rates of *sliaa9* mutants and the wild-type under low light conditions. **(B)** Shoot growth rates of *sliaa9* mutants and the wild-type under normal light conditions. **(C)** Length of 3rd–10th internodes of the *sliaa9* mutants and the wild-type at day 48. **(D)** Hypocotyl length of young seedlings of *sliaa9* mutants and the wild-type at day 21. MM; Moneymaker, RG; Rio Grande, AC, Ailsa Craig. T2 lines from MM#2-13, RG#4-1, and AC#1-1 (T1 lines) were analyzed. Data are means ± S.D. of independent T2 plant lines (*n* = 3–6). low; grown in low light conditions, normal; grown in normal light conditions. ^*^p < 0.05, ^**^p < 0.01, and ^***^p < 0.001 are determined by Student’s *t* tests. n.s., not significant.

We next analyzed the effects of *SlIAA9* disruption on plant growth among cultivars under different light conditions: low (40–70 μmol m^−2^ s^−1^) or normal (150–250 μmol m^−2^ s^−1^) light intensities. MM-*sliaa9* and RG-*sliaa9* showed slower rates of shoot elongation growth in the early growth stages compared with wild-type under low light intensity, whereas AC-*sliaa9* did not show clear difference in growth rates (Figures 1D, 2A). Shoot lengths of wild-type and *sliaa9* mutants in Moneymaker and Rio Grande grown under normal light intensity were mostly longer than those grown under low light intensity, on the contrary, Ailsa Craig plants did not show large differences between light conditions (Figures 2A,B). Moneymaker *sliaa9* showed a strong phenotype in the internode elongation, and this phenotype of Rio Grande mutant was apparent in low light intensity (Figure 2C). On the contrary, there were no significant differences in the internode length of Ailsa Craig *sliaa9* and wild-type under the different light intensity (Figure 2C). These results suggest that *sliaa9* mutants showed different shoot elongation growth phenotypes among cultivars.

Next, we analyzed the effect of light intensity on hypocotyl growth of young seedlings of tomato *sliaa9* mutants grown in different light intensity to identify the AUX/IAA9 function in light response in the tomato cultivars. The results indicated a different response to light conditions among mutants of the three cultivars (Figure 2D). Thus, the hypocotyl length of MM-*sliaa9* was smaller than that of wild-type under both light intensity, whereas, AC-*sliaa9* showed a longer hypocotyl than that of wild-type in low light intensity; however, the effect on hypocotyl growth was rescued in normal light, and hypocotyl length of mutant and wild-type were similarly decreased (Figure 2D). There was no clear differences between RG-*sliaa9* and wild-type; hypocotyl length of mutant and wild-type were similarly elongated under low light intensity (Figure 2D). Furthermore, hypocotyl elongation in Moneymaker is strongly affected by *SlIAA9* knockout and did not show any further effects of light intensity. These results might suggest that photomorphogenesis in the hypocotyl elongation of young seedlings differs in different tomato cultivars, and the relationship of the auxin signaling pathway mediated by SlIAA9 and photomorphogenesis might be clearer in Ailsa Craig than in Moneymaker and Rio Grande.

To understand molecular mechanisms of elongation growth controlled by SlIAA9 and the downstream pathway, we focused on the expression of *SlIAA3* gene. The *SlIAA3* expression levels were analyzed in the third internodes and hypocotyls of young seedlings grown under low or normal light intensity (Supplementary Figure S3). The results indicated that there were not close relationship of the *SlIAA3* expression levels, elongation growth mediated by SlIAA9, and the light intensity among cultivars, although weak downregulation of the *SlIAA3* expression was observed in the *SlIAA9* Rio Grande mutants. The results suggest that complicated pathways might be existed in the downstream of SlIAA9 in elongation growth of the tomato cultivars.

### Leaf Morphology of Tomato *sliaa9* Mutants

**Figure 3:**
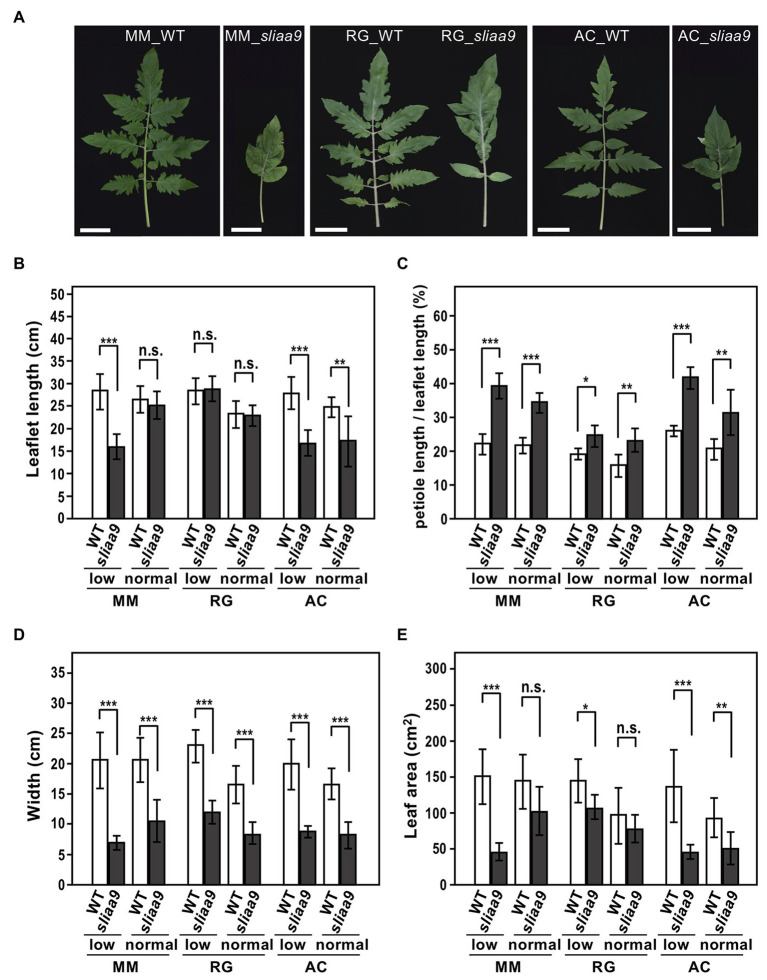
Altered leaf morphology of *sliaa9* mutants in three tomato cultivars. **(A)** Leaf morphology of *sliaa9* mutants and wild-type plants grown under low light conditions. Bars = 5cm. **(B–E)** Leaflet length **(B)**, the ratio of petioles to total leaf length **(C)**, leaf width **(D)**, and leaf area **(E)** in *sliaa9* plants and the wild-type. T2 lines from MM#2-13, RG#4-1, and AC#1-1 (T1 lines) were analyzed. Data are means ± S.D. of 2–5 leaves of individual T2 plant line (total 2–4 lines; *n* = 6–10). ^*^p < 0.05, ^**^p < 0.01, and ^***^p < 0.001 are determined by Student’s *t* tests. n.s., not significant.

We next analyzed the leaf morphology of tomato *sliaa9* mutants. Previous studies had shown that disruption of *SlIAA9* in tomato led to altered leaf morphology, with the compound leaf changing to a single leaf (Wang et al., 2005; Berger et al., 2009; Saito et al., 2011; Ueta et al., 2017). In the present study, the *sliaa9* mutants in the three cultivars exhibited morphological changes to the single leaf (Figure 3A). To analyze the phenotypes in elongation growth in leaves, we measured several parameters of fully expanded leaves in young plants (Supplementary Figures S4, S5; Figures 3B–E). The length of *sliaa9* mutant leaves in Moneymaker and Rio Grande showed no significant differences when compared with those of wild-type in normal lights, whereas those of the Moneymaker mutant in low light conditions and the Ailsa Craig mutant in both light conditions were shorter than those of wild-type (Figures 3A,B), indicating different mechanisms related to elongation in leaves and shoots between cultivars. When the ratio of petiole length to full length of leaves was compared among the mutants, all three cultivars exhibited a higher ratio in *sliaa9* mutants compared with wild-type in both light conditions (Figure 3C). Leaf width in *sliaa9* mutants of all three cultivars was narrower than that of wild-type, and the mutants of Moneymaker and Ailsa Craig also showed a smaller leaf area than wild-type especially in low light conditions (Figures 3D,E). These results suggest that *SlIAA9* knockout affected the several parameters in leaf shapes that might be differentially controlled in various tomato cultivars.

To analyze any further effects of *SlIAA9* disruption on plant growth, we measured leaf numbers and chlorophyll content in mature leaves of mutant plants using a Soil Plant Analysis Development (SPAD) chlorophyll meter. Leaf numbers increased similarly in *sliaa9* mutants and the wild-type of all three cultivars growing under both light conditions (Supplementary Figure S5). There were no significant differences in SPAD values between *sliaa9* mutants and the wild-type plants, except that Moneymaker plants showed larger values under normal light than in low light conditions (Supplementary Figure S4E).

### Parthenocarpy Phenotypes in *SlIAA9* Mutants

**Figure 4:**
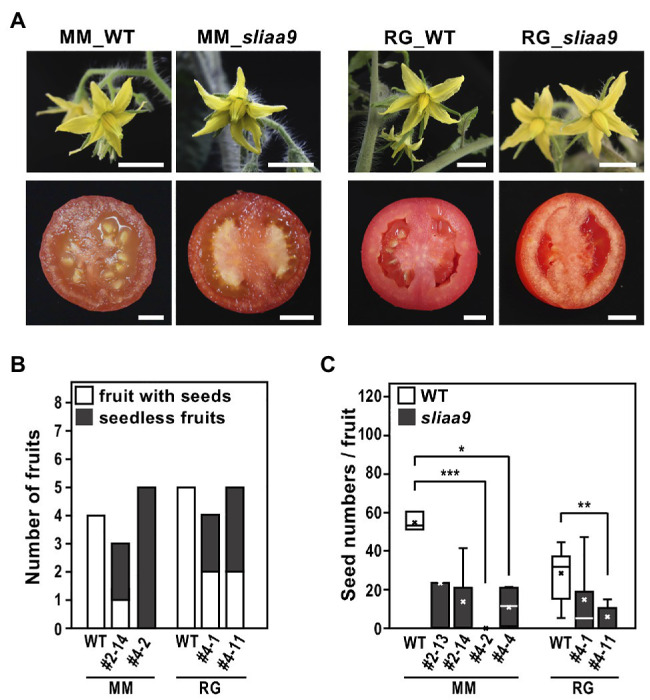
Flowers and fruits phenotypes of *sliaa9* mutants. **(A)** The representative T2 flower (upper, MM_*sliaa9*; MM#2-13-1, RG_*sliaa9*; and RG#4-1-1) and T0 fruit (bottom, MM_*sliaa9*; MM#4, RG_*sliaa9*; and RG#4) phenotypes of the *sliaa9* mutants of Moneymaker (left) and Rio Grande (Right). Bars = 1 cm. **(B)** Number of seedless mature fruits developed without pollination (parthenocarpic fruits) and all the fruits obtained from *sliaa9* T1 mutant lines (MM#2-14, MM#4-2, and RG#4-1, and RG#4-11) grown under low light for 5 months. **(C)** Seed numbers of *sliaa9* T1 mutant lines (MM#2-13, #2-14, #4-2, and #4-4) of Moneymaker and Rio Grande (RG#4-1, and #4-11). Average seed numbers were calculated in the mutant fruits (*n* = 3–12) without pollination and in wild-type (WT) fruits (*n* = 5–12) with pollination. ^*^p < 0.05, ^**^p < 0.01, and ^***^p < 0.001 are determined by Student’s *t* tests.

We next analyzed flower and fruit phenotypes of the *sliaa9* mutants of Moneymaker and Rio Grande. Flower development in all these mutants showed no differences when compared with wild-type plants (Figure 4A). Fruit shape in *sliaa9* mutants was also similar to that of the wild-type; on the contrary, several fruits had no, or only few, seeds, suggesting a parthenocarpic phenotype as shown in previous studies using cultivars Micro-Tom and Ailsa Craig (Figure 4A; Ueta et al., 2017). The total number of fruit, and seed numbers, were measured 2 months after the first fruit was fully ripened, indicating that parthenocarpic fruits were generated only in *sliaa9* mutants (Figures 4B,C). These results indicate that the role of SlIAA9 in fruit development is common to the three cultivars, and that parthenocarpic phenotypes can be efficiently introduced into various cultivars by CRISPR-Cas9 technology.

## Discussion

Disruption and repression of *SlIAA9* – a negative regulator of the Aux/IAA transcription factor – has been reported to induce parthenocarpy in tomato cultivars Micro-Tom and Ailsa Craig and also changes in leaf morphology and plant form in several more cultivars, including Micro-Tom, Ailsa Craig, M82, and Red Setter (Wang et al., 2005; Zhang et al., 2007; Berger et al., 2009; Saito et al., 2011; Mazzucato et al., 2015; Ueta et al., 2017). Several other phenotypes in *SlIAA9* antisense lines of Micro-Tom (*AS-IAA9*) have also been reported, e.g., hypersensitivity to auxin, enhanced hypocotyl/stem elongation, and reduced apical dominance were observed in *AS-IAA9* plants (Wang et al., 2005); however, few studies have compared *SlIAA9* knockout phenotypes in various tomato cultivars. In this study, *sliaa9* mutant lines of various commercial tomato cultivars were generated using CRISPR-Cas9 technology. Null-segregants of *sliaa9* knockout mutants (progenies of MM#2, RG#4, and AC#1), were isolated and plant phenotypes – shoot elongation, leaf morphology, and parthenocarpy – were analyzed using the mutants grown under the different light conditions.

**Figure 5:**
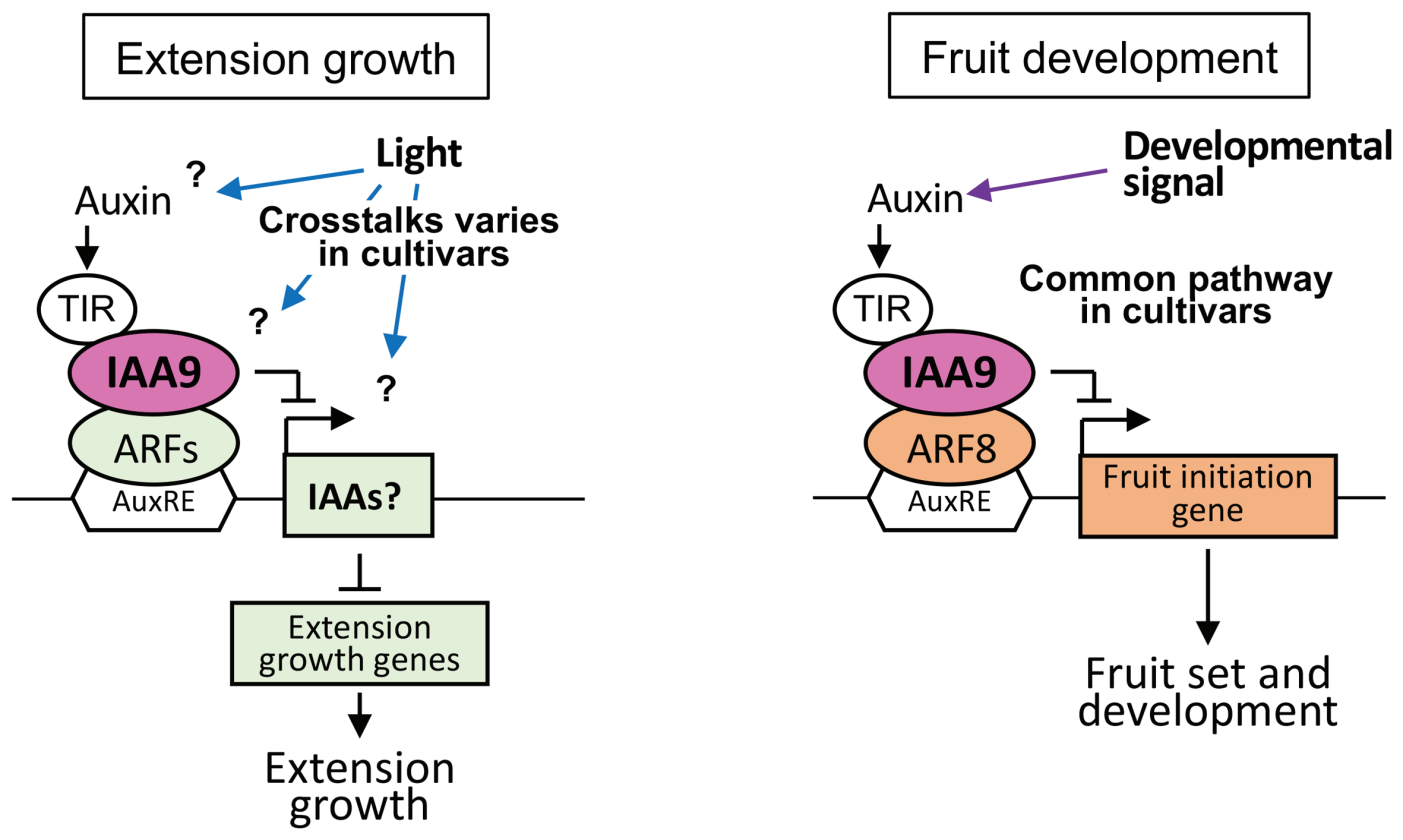
Control mechanisms of elongation growth and fruit development mediated by *SlIAA9* in various tomato cultivars. (**Left**) A hypothetical model of shoot elongation controlled by SlIAA9 in tomato cultivars, Moneymaker, Rio Grande, and Ailsa Craig. (**Right**) A model of fruit development commonly controlled by SlIAA9 in three tomato cultivars.

The elongation of shoots and internodes in cultivars Moneymaker and Rio Grande were commonly decreased in *sliaa9* mutants in low light conditions. Although elongation growth were increased through enhanced auxin signaling following *SlIAA9* disruption in the previous studies (Wang et al., 2005), the opposite effect on shoot elongation was detected in these cultivars. Elongation growth patterns in leaves of mutants were also different among these two cultivars, as similar to shoot elongation phenotypes. Previous studies had shown that *AS-IAA9* (*SlIAA9* antisense line) in Micro-Tom exhibited an enhanced stem/hypocotyl elongation growth phenotype (Wang et al., 2005), and the gain-of-function mutant of *SlIAA9* with a single amino acid substitution reduced the elongation of internodes and showed a strong dwarf phenotype in cv. VF36 (Koenig et al., 2009). These results suggested the existence of different mechanisms of auxin signaling among tomato cultivars and leaf and shoots under different environmental conditions (Figure 5). Double knockout mutants of *Arabidopsis IAA8* and *IAA9* genes, which belong to the same clade as *SlIAA9*, exhibited no differences other than leaf morphology between wild-type plants (Overvoorde et al., 2005; Koenig et al., 2009), suggesting that SlIAA9 function in leaf morphogenesis also varied among plant species.

We analyzed the effects of light intensity on plant stem and leaf growth of the various tomato cultivars and the relationship with *SlIAA9* disruption. Interestingly, the results indicated that SlIAA9 function in stem elongation growth varied among cultivars and light conditions, with Rio Grande showing the enhanced growth of young seedlings; this was even more apparent in the *sliaa9* mutant of Aila Craig. In *Arabidopsis*, it has been shown that light conditions affect the auxin signal pathway, biosynthesis, and transport system (Halliday et al., 2009). Under high light conditions, PHYB and CRY1 are activated and inhibit the AUX/IAA degradation, resulting in the inhibition of expression of auxin responsive genes for elongation growth (Ma and Li, 2019). Under low light or low F:FR conditions, transcription of auxin responsive genes is upregulated through the activation of PIF4/5/7, and elongation growth is enhanced in *Arabidopsis* (Ma and Li, 2019). Tomato cultivar M82 and wild relatives of tomato (e.g., *Solanum pennellii*) exhibited shade avoidance responses (e.g., hypocotyl and petiole elongation) under shade or dim-light conditions (Chitwood et al., 2015), suggesting that the mechanisms of elongation growth under light conditions of these tomatoes might be similar with those in Rio Grande.

The upregulation of *SlIAA3* – an auxin responsive gene (Chaabouni et al., 2009) – has been reported in *SlIAA9* knockout plants, Ailsa Craig *entire* LA2922 and Micro-Tom *AS-IAA9* (Wang et al., 2005; Zhang et al., 2007). The Micro-Tom *AS-IAA3* line (*SlIAA3* antisense line) showed decreased auxin responses and reduced hypocotyl elongation (Chaabouni et al., 2009). In the present study, AC-*sliaa9* hypocotyls were longer than those of wild-type under low light conditions, suggesting that upregulation of *SlIAA3* might have occurred in this mutant (Wang et al., 2005). In this study, the *SlIAA3* expression levels is not strongly related to the mutant phenotypes for elongation growth and response to light intensity except Rio Grande and more complicated pathways might be existed in the downstream of IAA9 in elongation growth of the tomato cultivars (Figure 5). Further detailed studies at the molecular level are needed to clarify the regulatory mechanisms involved, including the crosstalk of auxin and light signal transduction in tomato cultivars.

The *sliaa9* mutants generated by CRISPR-Cas9 in the various commercial cultivars used here showed similar parthenocarpic phenotypes without any changes in fruit yield, suggesting the usefulness of these mutant lines for agricultural applications. Our results suggest that the function of SlIAA9 in fruit development is conserved among tomato varieties, although shoot growth varied, and that these phenotypic changes are also affected by environmental factors, including light conditions. Our results add to the accumulating studies using novel mutants created by genome editing in useful cultivars that will further contribute to new findings in plant functions for further application of environmental control in agriculture.

## Data Availability Statement

The raw data supporting the conclusions of this article will be made available by the authors, without undue reservation.

## Author Contributions

YO, KO, and CA-H wrote the manuscript. CA-H performed most of the experiments and analyzed the data. KY and NW performed the genetic analysis. RU and RH designed the gRNA and transformation. KO supervised the research. YO designed, led, and coordinated the overall study. All authors contributed to the article and approved the submitted version.

### Conflict of Interest

The authors declare that the research was conducted in the absence of any commercial or financial relationships that could be construed as a potential conflict of interest.
