# Supplementary material for: Effects of the sliaa9 Mutation on Shoot Elongation Growth of Tomato Cultivars
Source: Front Plant Sci. 2021 May 20;12:627832. doi: 10.3389/fpls.2021.627832 (PMC8173035; doi:10.3389/fpls.2021.627832)
Supplement: Supplementary file 1 [file Data_Sheet_1.pdf]

Table S1. Primers used in this study.		
primer name	sequences (5'- 3')	PCR products
**PCR-RFLP of the target region for *SlIAA9***		
SlIAA9_F27-52	GGAGGAGGAGGGCCAGAGTAATGTAA	349 bp
SlIAA9_R375-348	GTTGCCACTAACTACTGTTTTCTGCGAT	
**Cloning of the target region by SLiCE method**		
SLiCE_IAA9_F27-52	ACCCGGGGGCGCGCCGGATCGGAGGAGGAGGGCCAGAGTAATGTAA	389 bp
SLiCE_IAA9_R375-348	TCTAGACTTAATTAAGGATCGTTGCCACTAACTACTGTTTTCTGCGAT	
**Generation of DIG-Labeled Probes for Southern blot**		
Southern_Probe_gRNA_Fw	AAGCTTCGTTGAACAACGGAAAC	624 bp
Southern_Probe_gRNA_Rv	AACGAAGAGAAAACCCCAGAAAT	
Southern_Probe_AtCas9_Fw	GCAGCTCAAAGAGGATTACTTCA	481 bp
Southern_Probe_AtCas9_Rv	CTCATGGAGACTATCACCCTGTC	
Southern_Probe_LHCB_Fw	GGTGAATTCCCTGGTGACTACGGGTG	430 bp
Southern_Probe_LHCB_Rv	TCTCCTTTACCTTGAGCTCAGCAAA	
**1st PCR primer for the off-target in next-generation sequencing**		
MiSeq500_IAA9_off1_Fw	ACACTCTTTCCCTACACGACGCTCTTCCGATCTAGTTCTTTTTGTTGTTCTCAGCATC	487 bp
MiSeq500_IAA9_off1_Rv	GTGACTGGAGTTCAGACGTGTGCTCTTCCGATCTGTAGATTTGGACAGTTCTTTGAAGC	
**PCR detection of T-DNA regions of CRISPR-Cas9 expression vector**		
M13Uni-100	GATCGGTGCGGGCCTCTTCGCTATT	① RB-AtU6-26, 209bp
AtU6-26_+62Rv	GCTAAGAAGAAATGATGTATTGTGC	
Southern_probe_gRNA_Fw	AAGCTTCGTTGAACAACGGAAAC	② gRNA, 624bp
Southern_probe_gRNA_Rv	AACGAAGAGAAAACCCCAGAAAT	
gRNA_seqF2	GTTTTAGAGCTAGAAATAGCAA	③ gRNA-pro-Cas9, 1150bp
At_Cas9_115-96Rv	CGGTGTTTCCGAGAACCTTG	
Southern_probe_AtCas9_Fw	GCAGCTCAAAGAGGATTACTTCA	④ Cas9, 481bp
Southern_probe_AtCas9_Rv	CTCATGGAGACTATCACCCTGTC	
AtCas9_Seq3600_F	ATCTCATCATCAAGCTCCCAAAG	⑤ Cas9-NLS-2A-GFP, 735bp
GFP Nter SeqR1	TGAACAGCTCCTCGCCCTTGCTCA	
GFP_Nter_seqF1	GTGAGCAAGGGCGAGGAGCTGTTCA	⑥ GFP, 824bp
T18.2_seqR1	AAGCCACAAATTCATAACACAACAAGCCA	
T18.2_seqF2	TGGCTTGTTGTGTTATGAATTTGTGGCTT	⑦ ter-Km, 1511bp
2301-Km_seqR1	TTCGCCCAATAGCAGCCAGTCCCTT	
35S-58	CCCACTATCCTTCGCAA	⑧ Km, 935bp
2301-Km_seqR2	GTCCCGCTCAGAAGAACTCGTCAAGA	
2300-Km_SeqF3	CGCTATCAGGACATAGCGTTGGCTAC	⑨ Km-LB, 621 bp
LB_-68Rv	TTAATGTACTGAATTAACGCCGAAT	

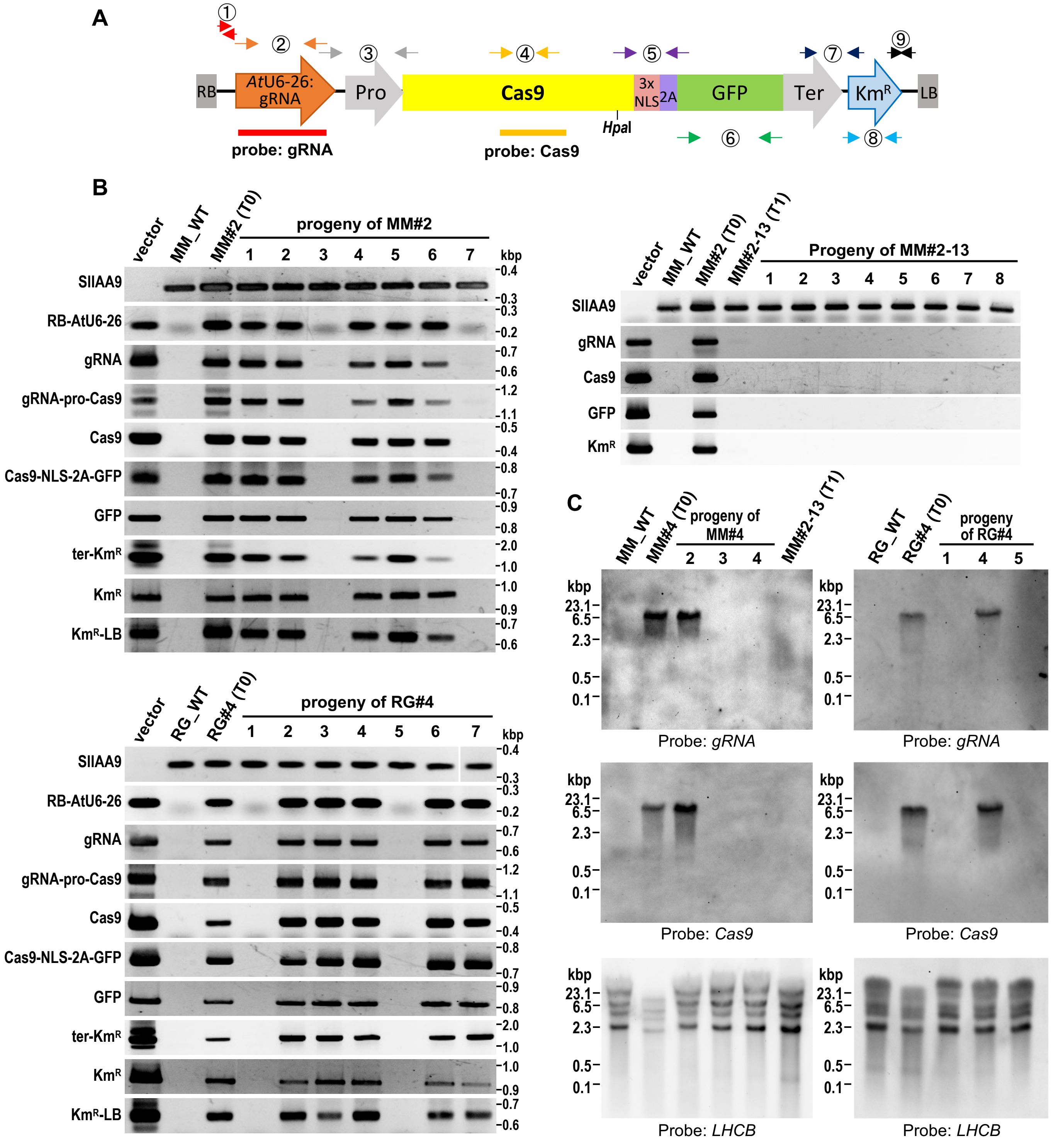

Figure S1. Isolation of null-segregants in the T1 and T2 progeny of *sliaa9* mutants.

(A) Structure of T-DNA regions of CRISPR-Cas9 expression vector and primer sites. In the pEgP237-2A-GFP vector, the 2 × *CaMV35S* promoter with omega enhancer sequence was used for *Cas9* expression. U6-26: Arabidopsis *U6 snRNA-26* promoter, gRNA: gRNA sequence, AtCas9; codon-optimized SpCas9 for *Arabidopsis thaliana*, NLS: nuclear localization signal, 2A; 2A self-cleavage peptide, Ter: Arabidopsis *hsp18.2* terminator, Km^R; kanamycin resistance gene *NPTII*, RB: right border of T-DNA, LB: left border of T-DNA. Arrows with numbers represent the PCR primer locations for detection of T-DNA regions in *sliaa9* mutants. The probe regions for *gRNA* and *Cas9* used in Southern blot analysis are also indicated. ①; RB-AtU6-26, ②; gRNA, ③; gRNA-pro-Cas9, ④; Cas9, ⑤; Cas9-NLS-2A-GFP, ⑥; GFP, ⑦; ter-Km^R, ⑧; Km^R, ⑨; Km^R-LB.

(B) Detection of T-DNA insertion in *sliaa9* T1 (MM#2-1 - MM#2-7 and RG#4-1 - RG#4-7) **(left)** and T2 (MM#2-13-1 - #2-13-8) **(right)** plants by PCR. MM#2, RG#4, and MM#2-13 are their parental plants, respectively. Each primer set is shown in **(A)** and **Table S1**. MM; Moneymaker, RG; Rio Grande. The *SlIAA9* gene was used as an endogenous control. # numbers; individual lines.

(C) Southern blot analysis was performed to isolate null-segregants of *sliaa9* mutants. DIG-labeled *gRNA* and *Cas9* probes were used to detect T-DNA insertion in *Hpa*I-digested genomic DNA of *sliaa9* mutants. The *LHCB* gene was used as an endogenous control.

```
On target:  GAGCTCAGGCTCGGTCTACCTGG
Off target: TATATCATGCTCGGTCTGCCAGG
```

Line No.	mutation frequencies*
MM_WT	11/11700 (0.09%)
MM#2 (T0)	12/10379 (0.12%)
RG_WT	2/1396 (0.14%)
RG#4 (T0)	2/2594 (0.08%)
RG#4-1 (T1)	3/3013 (0.10%)
RG#4-4 (T1)	2/3162 (0.06%)

*mutation efficiencies were calculated as mutation reads counts / total read counts.

Figure S2. Off-target mutation frequencies in the *sliaa9* mutants.
An off-target candidate sequence with 5 mismatches to the on-target sequence (chr6: 26946923–26946946) was analyzed to evaluate off-target effects in *sliaa9* mutants. The amplicon sequences were analyzed using MiSeq. Underlined; PAM, red; mismatching bases.

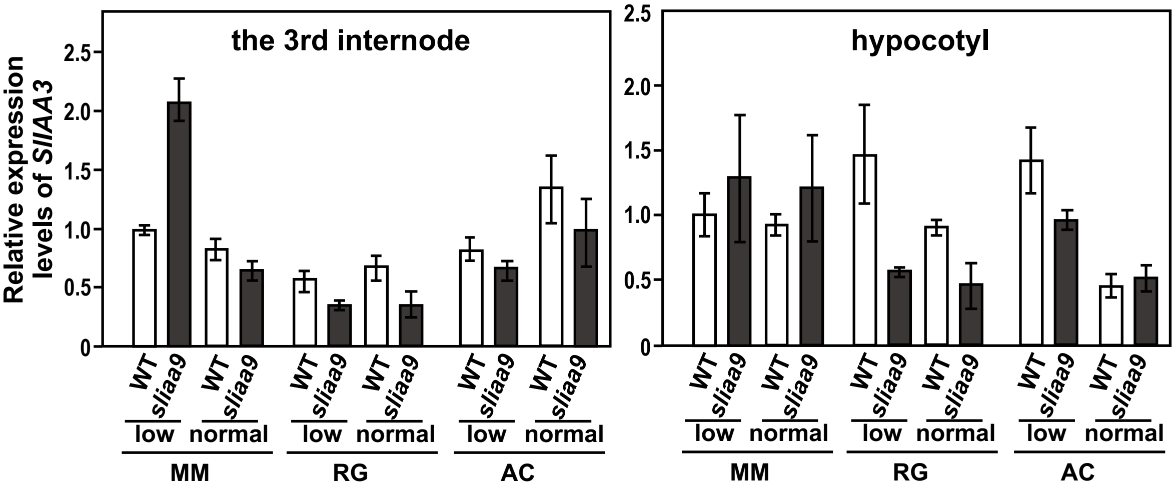

Figure S3. Expression levels of the *SlIAA3* gene in the *sliaa9* mutants.
The expression levels of *SlIAA3* were determined in the mutant tomato seedlings grown under low or normal light intensity. The relative expression levels were by normalized relative to those of a reference gene, *Slactin7-like*.

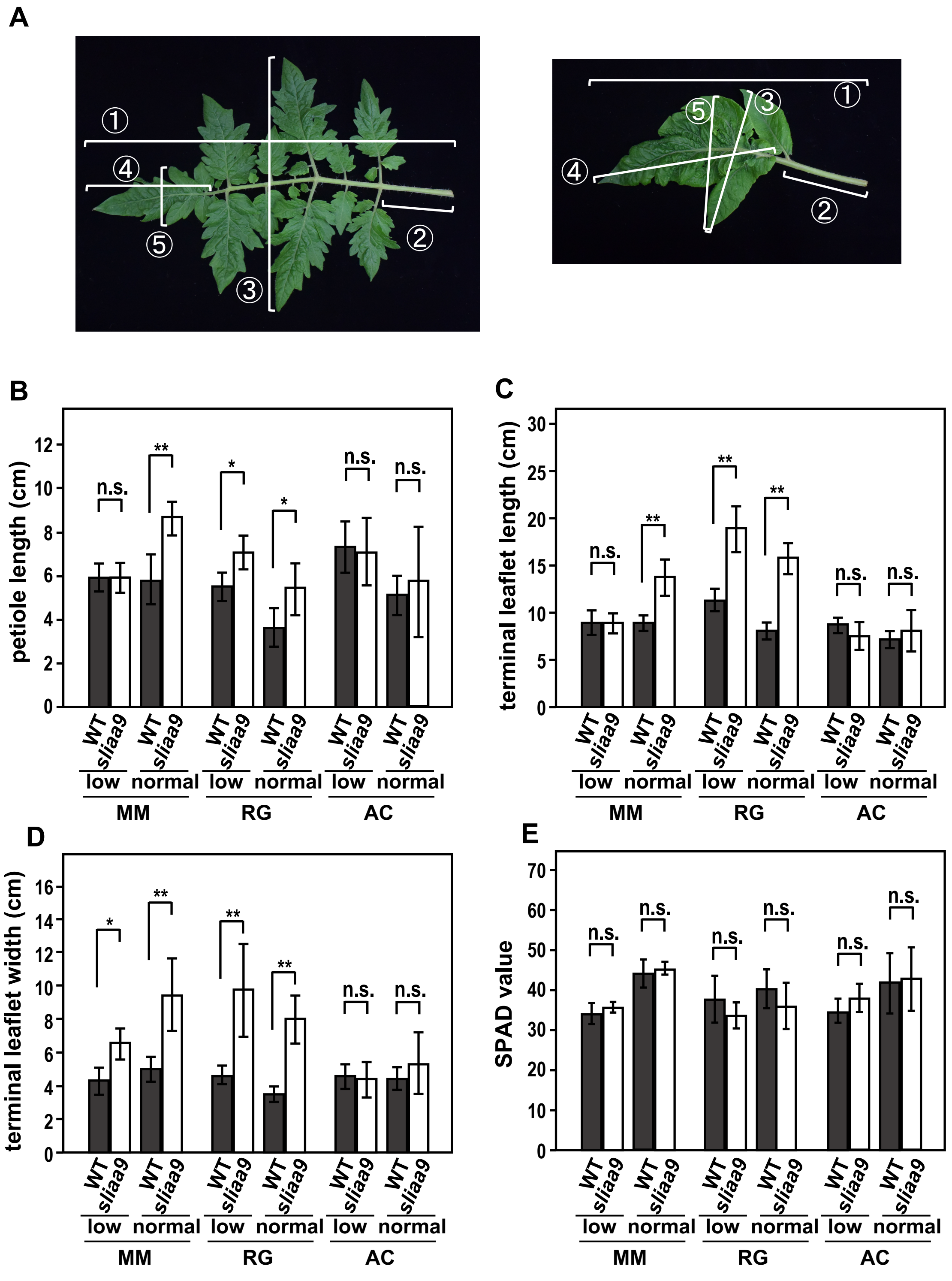

Figure S4. Measurement of leaf parts.

(A) Wild-type (left) and the *sliaa9* mutant (right) tomato leaves and the positions of various parameter of the leaves. ①; leaflet length, ②; petiole length, ③; leaf width, ④; terminal leaflet length, ⑤; terminal leaflet width.

(B-D) Petiole length **(B)**, terminal leaflet length **(C)**, and terminal leaflet width **(D)** were measured in leaves of *sliaa9* mutants and the wild type. The fifth to tenth fully expanded leaves of the mature plants were measured at 48 days after germination. Data are means ± S.D. of 2 – 5 leaves of individual T2 plant line (total 2 – 4 lines) (n = 6 – 10). n.s.; not significant.

(E) SPAD values of the *sliaa9* mutant and wild-type leaves. Data are means ± S.D. of independent plants (n = 20 - 30). *$P < 0.01$, and **$P < 0.001$ are determined by Student's t tests. n.s.; not significant.

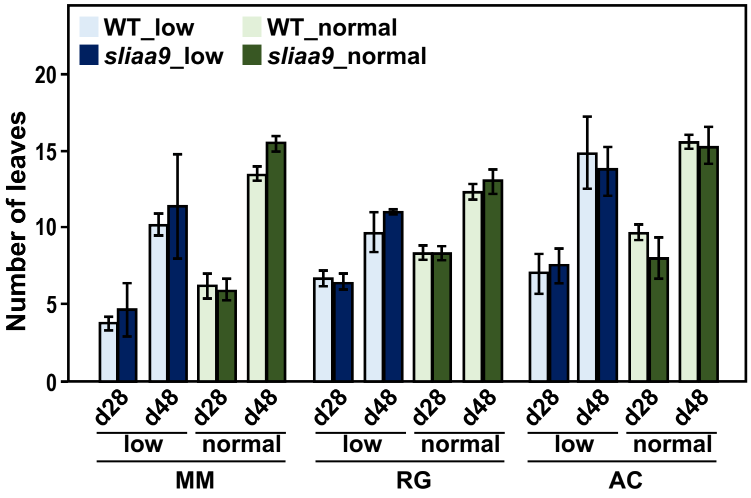

Figure S5. Leaf numbers of *sliaa9* mutants and wild-type during growth under different conditions.

Leaf numbers were measured in the *sliaa9* mutants and wild-type plants grown under low light and normal light conditions at 28 and 48 day after germination. Data are means ± S.D. of independent plants (*n* = 3-6).
